# Cardiac function in pregnant women with preeclampsia

**DOI:** 10.3389/fcvm.2024.1415727

**Published:** 2024-12-16

**Authors:** Gülen Yerlikaya-Schatten, Eva Karner, Florian Heinzl, Suriya Prausmüller, Stefan Kastl, Stephanie Springer, Robert Zilberszac

**Affiliations:** ^1^ Department of Obstetrics and Gynecology, Division of Obstetrics and Feto-maternal Medicine, Medical University of Vienna, Vienna, Austria; ^2^ Department of Cardiology, Medical University of Vienna, Vienna, Austria

**Keywords:** preeclampsia, NT-proBNP, maternal echocardiography, left ventricular hypertrophy, hypertension

## Abstract

**Introduction:**

Preeclampsia (PE) is thought to be the consequence of impaired placental perfusion leading to placental hypoxia. While it has been demonstrated that PE may be a consequence of maternal cardiovascular maladaptation, the exact role of maternal cardiac function remains to be determined. This study sought to assess cardiac characteristics in pregnant women diagnosed with PE and to determine the possible relationship between PE, maternal cardiac changes/function, and NT-proBNP levels.

**Methods:**

This was a retrospective analysis of 65 pregnant women diagnosed with PE who had an echocardiographic examination during pregnancy. Where available, NT-proBNP levels were analyzed. All patients underwent a comprehensive echocardiographic examination based on a standardized examination protocol.

**Results:**

Left ventricular size was within the normal range, and there was normal radial left ventricular function. Longitudinal contractility was impaired with a global longitudinal strain of −17.8% (quartiles −20.2 to −15.4). The cardiac index was in the normal range with a median of 3.2 ml/min/m^2^ (quartiles 2.6–4.0). The left atrium was of borderline size in longitudinal diameter [50 (44.8–54.3) mm], but within the normal range in volumetric index [27.3 (22.9–37.3) ml/m^2^]. Furthermore, mild left ventricular hypertrophy [septal thickness 12 (10–13) mm] and at least borderline elevated filling pressures with an E/e' ratio of 10.6 (8.5–12.9) were found. Maximal tricuspid regurgitation velocity [2.9 (2.5–3.3) m/s] and derived systolic pulmonary pressure [38 (29.5–44.5) mmHg] were borderline elevated. Regarding NT-proBNP levels, an increase in NT-proBNP levels correlated with a decrease in gestational age at delivery (*p* < 0.0002) and maternal cardiac changes. Obstetric characteristics showed a preterm rate of 71.43%, mostly due to maternal aggravation of PE or because of fetal signs of deprivation based on placental insufficiency. Neonatal deaths occurred in five cases (7.69%).

**Conclusion:**

Changes in cardiac function in the context of hypertensive pregnancy diseases can be observed with regard to various echocardiographic parameters. Furthermore, there is a significant association between NT-proBNP levels and a decrease in gestational age at delivery in women with PE, which thus might be useful as a prognostic factor for the management of women with preeclampsia and changes in maternal cardiac function during pregnancy.

## Introduction

Preeclampsia (PE) is thought to be the consequence of impaired placental perfusion leading to placental hypoxia. PE is characterized by maternal hypertension and at least one associated end-organ dysfunction with or without proteinuria, as well as cardiovascular complications. The incidence of PE is 5%–7% of all pregnant women (1–4). The specific underlying pathomechanism has not yet been clarified, but it has long been established that PE is caused by impaired placental perfusion due to placental hypoxia (5, 6).

It has been demonstrated that PE may be a consequence of maternal cardiovascular maladaptation, but the exact role of maternal cardiac function remains to be determined (7, 8). One hypothesis suggests that impaired remodeling of spiral arteries during placentation, potentially due to a predisposition to cardiovascular dysfunction, may result in narrow placental arterioles, leading to elevated blood pressure in upstream vessels, particularly noticeable in the uterine arteries. The remodeling process is intricate and not fully understood, involving immune cell activation and the secretion of angiogenic growth factors to create high-flow, low-resistance arterioles. An imbalance in these factors can disrupt maternal endothelial function and contribute to hypertension. There is ongoing discussion about whether preeclampsia may originate from a placental or cardiovascular condition.

While it has been demonstrated that developing PE in pregnancy is associated with a higher risk of developing cardiovascular disease in later life (9), previous studies have reported mixed results regarding maternal cardiac changes during pregnancy complicated by PE, mostly focusing on left ventricular function (3, 4, 10–12). In PE, changes in several maternal cardiac parameters have been reported, among them left ventricular mass and filling pressures, as well as left ventricular diastolic dysfunction, reduced cardiac index, and reduced venous reserve capacity (3, 4, 8, 10–12).

In addition to transthoracic echocardiography, neurohormones, specifically aminoterminal pro-brain natriuretic peptide (NT-proBNP) can be used for cardiac function assessment and the detection of heart failure (13). The diagnostic utility of this marker in various conditions such as gestational hypertension (GH), PE, and gestational diabetes is yet to be firmly established. Furthermore, defining normal reference values during pregnancies and elucidating potential clinical implications and interpretations of serum level elevation exhibit considerable variations. Franz et al. compared NT-proBNP levels in healthy pregnant and non-pregnant women and observed that values were higher in pregnant women than in controls and that they were subject to a dynamic process (14). Chang et al., on the other hand, demonstrated that NT-proBNP remains steady over the course of pregnancy of stable cardiac patients. However, they concluded that NT-proBNP levels above 200 pg/ml are independently associated with heart failure and preeclampsia and may be used as a prognostic/predictive factor (15).

The objectives of this study were twofold: firstly, to delineate the echocardiographic characteristics observed in pregnant women diagnosed with preeclampsia (PE) and, secondly, to explore the potential relationships between PE, maternal cardiac function changes, and NT-proBNP levels. Our findings aim to generate hypotheses that could inform the development of future risk prediction models. These models may potentially predict the severity of preeclampsia, characterized by outcomes such as preterm delivery due to preeclampsia, and postpartum maternal and neonatal outcomes.

## Methods

### Patient population

This was a retrospective analysis of prospectively collected data in women developing preeclampsia during pregnancy in one tertiary referral center.

Sixty-five patients were included in the study, all of whom had maternal cardiac echocardiography during pregnancy. Where available NT-proBNP and SFLT/PLGF were collected and evaluated. NT-proBNP serum levels, described as pg/ml were obtained by a quantitative *in vitro* test based on electrochemiluminescence immunoassays (ECLIA). Therefore, the inclusion criteria were pregnant women with confirmed preeclampsia who had performed maternal echocardiography. Preeclampsia was diagnosed in women with new-onset hypertension in pregnancy (>20 weeks of gestation) and proteinuria or another preeclampsia-associated organ dysfunction (16). Patients were excluded if they had no cardiac echo during pregnancy or if preeclampsia was not clearly documented as well as confirmed maternal congenital heart disease. Pregnancy and birth characteristics were obtained. All clinical information was extracted from the patient's hospital records. Maternal and neonatal outcomes were analyzed. Blood pressures and weight recorded at the time of echocardiography were collected. Following the 2023 ESH hypertension guidelines (17), patients were instructed to monitor their blood pressure at home twice daily (morning and evening) using a validated, automated upper-arm cuff device, ensuring measurements were taken in a resting state without prior caffeine intake. In-clinic blood pressure measurements were conducted after a period of rest in a seated position, also using an automated upper-arm device. Perinatal outcome assessed, included gestational age at birth, live birth, APGAR score, and admission to the neonatal unit. The study protocol complies with the Declaration of Helsinki and was approved by the ethics committee of the Medical University of Vienna and was exempted from informed consent requirements owing to its observational study design**.**

### Echocardiographic data

All patients underwent a comprehensive echocardiographic examination based on a standardized examination protocol including two-dimensional echocardiography, conventional, tissue, and color Doppler by an experienced echocardiographer using GE Vivid E95 Echocardiography Systems, and the *post hoc* image assessment was carried out with GE EchoPAC Echocardiography Image Analysis Software [both GE Healthcare (Chicago, IL, USA)]. Mitral inflow velocities were measured at the leaflet tips by pulsed-wave (PW) Doppler. Tissue Doppler velocities were measured from the apical four-chamber view at the medial mitral annulus. Chamber size and function assessment was performed in accordance with current recommendations (18). The left atrial (LA) volumes were measured at end-ventricular systole and indexed to the body surface area (BSA). LA diameters were derived from apical four-chamber views measured at the end of systole, the diameter being measured parallel to the interatrial septum from the plane of the mitral annulus to the roof of the atrium (19).

The peak tricuspid regurgitation velocities were recorded in the apical four-chamber view, and systolic pulmonary pressure was quantified using the simplified Bernoulli equation. LV end-systolic and end-diastolic volumes were measured from apical four- and two-chamber views, and LVEF was calculated by the Simpson method. Global longitudinal strain (GLS) was obtained using endocardial contours from the apical four-chamber, apical two-chamber, and apical long-axis views, respectively, timed at one complete RR interval.

Doppler stroke volume (SV) was calculated as the product of the right ventricular (RV) outflow tract (RVOT) cross-sectional area and pulsed-wave Doppler velocity–time integral (VTI) recorded at the RVOT. A minimum of three cardiac cycles were recorded and averaged for analysis. The RVOT diameter was measured immediately proximal to the pulmonary valve annulus in the parasternal short-axis view, and RVOT VTI was measured using pulsed-wave Doppler in the parasternal short-axis view. SV index (SVI) was calculated by dividing SV by BSA, CO as the product of SV and heart rate, and cardiac index (CI) as CO indexed by BSA.

### Statistical analysis

Data are expressed as median (interquartile range) for numerical variables and absolute (relative) frequencies for categorical variables.

We used the Kendall tau test to compare the degree of correlation between the variables. Furthermore, non-parametric linear regression via Theil–Sen estimator was used to model the relationship of continuous variables, while the correspondence from continuous variables to binary ones was examined via logistic regression.

A *p*-value of <0.05 was set for statistical significance. Statistical analyses was performed using R (version 4.3.0) (20) and contributing packages mblm 0.12.1 (21), flextable 0.9.1 (22), tidyverse 2.0.0 (23), and viridis 0.6.3 (24).

## Results

### Obstetrics characteristics

The baseline patient characteristics are given in Table 1.

**Table 1:** Maternal characteristics and markers for preeclampsia.

Variable	*N* = 65	Data missing (%)
Age^1^	33 (30–38)	0 (0)
Body mass index (kg/cm^2^)^1^	29.4 (25–33)	12 (18.5)
Smoking^2^	6 (12)	15 (23.1)
Conception^2^		14 (21.5)
*In vitro* fertilization (IVF)	15 (29.41)	
Spontaneous	36 (70.59)	
NA	14 (21.54%)	
Nulliparity^2^	29 (44.62)	0 (0)
Gestational diabetes/diabetes mellitus^2^	17 (26.2)	0 (0)
Pre-existing hypertension^2^	12 (18.5)	0 (0)
Kidney disease^2^	3 (4.6)	0 (0)
Placental insufficiency^a^^2^	31 (47.69)	0 (0)
Early-onset PE^2^	47 (71.3)	0 (0)
PE with severe symptoms^2^	23 (35.4)	0 (0)
NT-proBNP serume level (pg/ml)^1^	414 (118–976)	18 (27.6)
Transfer to ICU^b^^2^	10 (15.38)	3 (4.6)
Hospital stay in days^1^	11 (7–16)	2 (3.1)
Systolic blood pressure (mmHg)^1^	163 (135–179)	3 (4.6)
Diastolic blood pressure (mmHg)^1^	100 (90–109)	3 (4.6)
Albumin	32.1 (29.4–34)	4 (6.2)
Sodium	137.00 (136.00–139)	5 (7.7)
Potassium	4.06 (3.81–4.35)	8 (12.3)
Chloride	104.00 (102.00–106)	5 (7.7)
Creatinin	0.70 (0.54–0.82)	5 (7.7)

Data presented as ^1^median (interquartile range) and ^2^number (percentage).

^a^ Placental insufficiency was defined as the low estimated fetal weight (<10.percentile) and pathological Doppler of uterine arteries or placental anomalies found by an ultrasound scan of an experienced placental sonographer.

^b^ ICU, intensive care unit.

Preeclampsia with severe symptoms was described as a birth indication in 23 patients (35.4%). Early-onset PE, defined as PE diagnosed before 34 weeks, was observed in 47 patients (71.3%). PE was diagnosed on average at gestational week 29 + 3. Overall, the gestational age at delivery was 32 weeks of gestation (quartiles 27–37 weeks) whereas 45 (71.4%) out of 65 women had a preterm delivery before 37 weeks due to preeclampsia. It is worth noting that preterm delivery was mainly due to maternal indication and not due to fetal decompensation, with rates of 64.1% and 34.4%, respectively. In 23 (35.4%) patients, severe preeclampsia was mentioned as an indication for preterm delivery. Worsening of PE led to preterm delivery and subsequent transfer to the intensive care unit (ICU) in 10 patients (15.4%) of the affected women.

NT-proBNP was collected and evaluated in 43 (66.2%) patients, whereas in 48 (73.84%) out of 65 women, sFLT-1 and PlGF as well as their ratio were obtained. The median serum level of sFLT-1 was 11,352.50 (quartiles 4,644.75–16,342), while the PlGF serum level was 66.58 (quartiles 26.74–143.74), providing a median serum level of 241.34 (quartiles 44–557) of the sFLT-1/PlGF ratio. Thirty-three patients (50.8%) showed elevated sFLT-1/PlGF ratio in the patients' serum. The cutoff for the prediction of PE was 85 before GW 34 and 110 after GW 34 regarding a high risk for PE within the next week. A sFLT-1/PlGF ratio under 38 was interpreted as low risk for PE within the next week (25).

**Figure 1:**
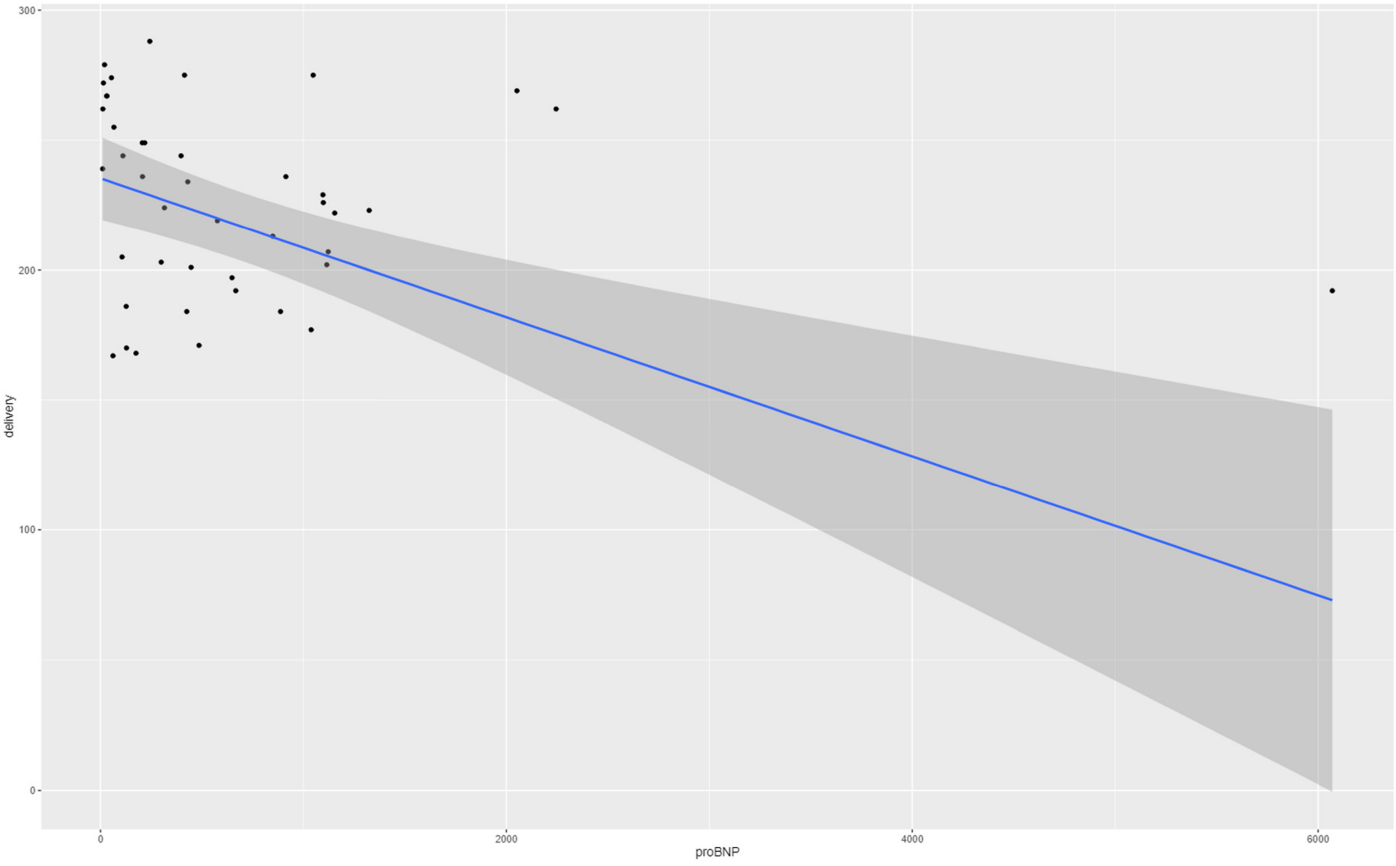
Gestational age at birth (in days) depending on NT-proBNP levels (pg/ml).

The medium NT-proBNP level in the maternal serum was 414 pg/ml (quartiles 118–976). Referring to the maternal cardiac changes and preeclampsia, a significant correlation between NT-proBNP and gestational age at delivery (*p* < 0.0002) was found via the Theil–Sen estimator with a coefficient of 0.0268. The correlation is presented in Figure 1. However, when correlated with sFLT-1/PlGF ratio, the analysis revealed a Kendall's tau of 0.365 (*p* = 0.001), which indicates no significant correlation. Furthermore, we tested the association of proBNP and systolic blood pressure values and diastolic blood pressure values, respectively, with Kendall's tau and observed pretty much no effect (tau = 0.023 with a p-value of 0.83 and tau = 0.037 with a *p*-value of 0.74, respectively).

### Postpartum maternal outcome

The average length of ICU stay was 3 days. The average hospital stay for our cohort was 11 days. Upon ICU admission, the patients were on an average of two antihypertensive agents. Antihypertensive therapy included methyldopa, uradipil, beta-blockers, and nifedipine. Beta-blockers used were metoprolol, bisoprolol, and in one case labetalol. Indication for postpartum ICU admission was mainly for hemodynamic monitoring, with none of the patients requiring mechanical ventilation or catecholamines. Five patients were treated with urapidil infusions due to systolic blood pressure levels of >190 mmHg. Four patients were transferred during pregnancy for maternal indications. One of those patients had preeclampsia with severe symptoms like dyspnea, complicated by a history of systemic lupus erythematosus, while the other three women presented with hypertensive crisis. All of those pregnancies were terminated per section during the hospital stay. One case of catastrophic antiphospholipid syndrome and IUFD (intrauterine fetal death) occurred in gestational week 22 + 3 presenting with massive hypertension, hemolysis, and acute kidney failure. The patient required dialysis and plasmapheresis and developed chronic renal failure requiring ongoing management.

### Neonatal outcome

The main reason for preterm delivery for fetal indication was fetal growth restriction (FGR) due to placental insufficiency. Overall, 31 patients (47.7%) were diagnosed with either FGR or a small for gestational age (SGA) baby. Most of these patients developed a severe course of growth restriction with highly pathologic Doppler flow velocities (58.1%; 18/31). The median fetal weight at birth was 1,450 (790–3,000) g [corresponding to median (IQR) 23rd (10–53) percentile], considering that 71.43% of the total cohort was delivered before 37 weeks of gestation.

Regarding neonatal outcome, 62 (96.9%) newborns were born alive, while three pregnancies ended in IUFD. Thirty-nine newborns (62.9%) required admission to the neonatal intensive care unit (NICU) for further treatment. One of them was admitted due to peripartum asphyxia, while all other newborns were transferred to the NICU due to prematurity ranging from 23 + 2 to 35 + 1 gestational age. In addition, most of these newborns were diagnosed with fetal growth restriction (69.23%; 22/39) and required respiratory support (51.28%; 20/39). Six had an intraventricular hemorrhage and four developed sepsis. The neonatal hospital stay ranged from a median of 5–155 days. In addition, organ malformations were described in four newborns, especially concerning the heart. Two newborns required heart surgery postnatally. All five neonatal deaths (7.7%) were caused by prematurity (gestational age 23 + 5–25 + 0) and were prenatally diagnosed with fetal growth restriction and born with extremely low birth weight (370–720 g) (Supplementary Table S1). Survival time was 3 days for one newborn after massive intraventricular hemorrhage, 4 days for another one with conatal infection, and 5 days for another newborn with sepsis and persistent fetal circulation. Two newborns survived almost one month and died because of necrotizing enterocolitis with intestinal perforation. For a description of neonatal outcome, see Table 2.

**Table 2:** Birth characteristics.

Variable	*N* = 65
Gestational age at birth^1^ (days)	223 (192–262)
Delivery per primary cesarean section^2^	49 (76.56)
Delivery per secondary cesarean section^2^	8 (12.5)
Birthweight^1^ (g)	1,450 (790–3,000)
Birthweight percentile^1^	23 (10–53)
APGAR of 5 min^1^	9 (9–10)
Umbilical pH	7.28 (7.24–7.31)
Delivery <37 weeks gestational age^2^	45 (71.43)
Livebirths^2^	62 (96.88)
Transfer to NICU*	39 (62.9)
Neonatal death	6 (9.52)
Invasive intervention	14 (22.58)

Data presented as ^1^median (interquartile range) and ^2^number (percentage).

^a^ NICU, neonatal intensive care unit.

### Echocardiographic characteristics

All 65 women underwent echocardiography at a median gestational age of 27 (quartiles 23–32) weeks. The median maternal age at that time point was 33 years (quartiles 31–40 years) with a median weight of 85 kg (quartiles 74–100 kg).

Left ventricular size was within the normal range with a volumetric index of 50.5 (41.9–55.4) ml/m^2^. There was normal radial left ventricular function [EF 60% (54.5–65.5)]; however, longitudinal contractility was impaired with a global longitudinal strain of −17.8% (−20.2 to −15.4). Cardiac index (cardiac output indexed to body surface area) was in the normal range with a median of 3.2 ml/min/m^2^ (quartiles 2.6–4.0). This was mainly due to the relatively high heart rates [median 80.5 (quartiles 72.5–91.0)], with a low normal stroke volume index of 42.0 ml/m^2^ (quartiles 31.3–51.7) The left atrium was of borderline size in longitudinal diameter [50 (44.8–54.3) mm], but within the normal range in volumetric index [27.3 (22.9–37.3) ml/m^2^]. Furthermore, mild left ventricular hypertrophy [septal thickness 12 (10–13) mm] and at least borderline elevated filling pressures with an E/e' ratio of 10.6 (8.5–12.9) were found. Maximal tricuspid regurgitation velocity [2.9 (2.5–3.3) m/s] and derived systolic pulmonary pressure [38 (29.5–44.5) mmHg] were borderline elevated. For summary of echocardiographic findings see Table 3.

**Table 3:** Echocardiographic findings.

Cardiac index (ml/m^2^)^1^	2.9 (2–4)	3 (4.6)
Stroke volume index (ml/m^2^)^1^	42 (31–52)	3 (4.6)
Left atrial longitudinal diameter (mm)^1^	49 (45–54)	2 (3.1)
Left atrial volume (ml)^1^	56 (44–72)	8 (12.3)
Left atrial volume index (ml/m^2^)	27.3 (22.9–37.3)	8 (12.3)
E/e’ ratio^1^	10.6 (8–13)	37 (57)
Left ventricular end-diastolic volume (ml)^1^	96 (81–114)	3 (4.6)
Left ventricular ejection fraction (%)^1^	60 (55–64)	16 (25)
Peak tricuspid regurgitation velocity (m/s)^1^	2.9 (2–3)	47 (72)
Systolic pulmonary artery pressure (mmHg)^1^	38 (30–45)	47 (72)
Left ventricular global longitudinal strain (%)^1^	−17.8 (−20.2–15.4)	24 (37)

Data presented as ^1^median (interquartile range) and ^2^number (percentage).

ICU, intensive care unit.

## Discussion

In this retrospective study, we assessed cardiac characteristics including echocardiographic and biochemical markers in pregnant women affected by preeclampsia. While our study is not the first to investigate the issue at hand, there is a remarkable paucity of stringent data, with a particular lack in recent years. A 2016 systematic review (26) identified 36 studies that included 745 women with GH and 815 women with preeclampsia from as early as 1982–2014.

Multiple observations were made in our study: We observed changes in several structural cardiac parameters such as borderline left atrial size and left ventricular hypertrophy. Furthermore, normal radial but limited longitudinal LV function, borderline elevated filling pressures, as well as borderline systolic pulmonary pressures were found. Strikingly, these pathological findings were diagnosed at a comparably early stage of pregnancy with a median gestational age of 27 weeks, indicating that women with early-onset preeclampsia are at particular risk of structural cardiac damage. In agreement with previous studies (26, 27), stroke volumes were in the low normal range, and cardiac output was maintained, mainly owing to tachycardiac heart rate regulation. Diastolic dysfunction, as expressed by an elevation of filling pressures by the E/e' ratio was present as described previously (28–32), particularly in early-onset preeclampsia (30); however, left atria were only mildly dilated when measured by diameters, but of normal dimension by volumetric measurement. This might be a reflection of the acuity of changes in filling pressures, given that the left atrium has been demonstrated to reflect not only severity but also chronicity of elevated LV end-diastolic pressures (33). LV hypertrophy, as reflected by intraventricular wall thickness, might be an earlier indicator of pressure overload and has been demonstrated to occur as early as 2 days after induction of pressure overload in animal models (34). Strikingly, LV hypertrophy has been demonstrated to be an independent predictor of adverse pregnancy outcomes (3). Further emphasizing the problem of elevated filling pressures, systolic pulmonary artery pressures, which too reflect diastolic dysfunction (35), were slightly elevated in our study. In agreement with previous data (12), subclinical LV dysfunction, as expressed by impaired global longitudinal strain, was present in our study, thus warranting clinical and echocardiographic follow-up after delivery to enable treating physicians to counteract the development of chronic heart failure.

ProBNP and its aminoterminal cleavage equivalent (NT-proBNP) have been established as biomarkers for heart failure and are therefore used in routine diagnostic testing for cardiac diseases (36). In pregnancy, the correlation between high levels of NT-proBNP and severe course of PE has been published (37). Garrido-Giménez et al. developed a predictive model including gestational age, chronic hypertension, sFlt1/PlGF ratio, NT-proBNP, and uric acid, which showed a better prediction of preterm PE compared to the use of sFlt1/PlGF ratio alone. However, NT-proBNP as a predictor for the development of PE has not been established yet (38). In addition, no standard levels have yet been described as reference cutoffs in pregnant women or women with PE. According to the ESC guidelines, NT-proBNP levels >125 pg/ml indicates an elevation, and acute heart failure has to be ruled out at a serum level > 300 pg/ml in non-pregnant patients (39). In our study cohort, high maternal NT-proBNP values indicate premature delivery and may reflect cardiac involvement in patients with PE. Therefore, patients with PE and elevated NT-proBNP levels are at higher risk for preterm delivery due to the development of severe symptoms and should be monitored regularly. In general, the median gestational age at delivery was 32 weeks of gestation in our patients. The main indication for preterm delivery was due to maternal aggravation of the condition associated with preeclampsia. These principal findings are supported by other studies: Giannubilo et al. presented in their cohort that NT-proBNP levels were higher in women with PE when compared with low-risk pregnancies (40). Another study observed that NT-proBNP levels above 200 pg/ml are independently associated with heart failure and PE and may be used as a predictive factor (15). In our collective, the medium serum level of NT-pro BNP was 414 pg/ml and therefore indicates the presence of preeclampsia with severe symptoms, which could be observed in 71.3% of our patients. It also describes a higher risk for cardiac decompensation. Cardiac decompensation in the sense of peripartum cardiomyopathy was not observed in our group. The question of whether echocardiography should be recommended in patients with preeclampsia to detect cardiac decompensation early is still to be determined. It is noteworthy, however, that an increase in NT-proBNP levels in uneventful pregnancies in comparison to non-pregnant women has been observed previously. Specifically, an increase up to about 23 weeks of gestation and then a decrease to levels that were comparable to non-pregnant women was demonstrated in that particular study (14).

Observing the obstetric characteristics of our collective, 71.5% of all patients required a preterm delivery due to the severity of preeclampsia. The median sFlt1/PlGF ratio was 240, which predicts a high risk of a severe course. Very high serum levels (>655) are associated with higher risks of obstetric complications such as iatrogenic preterm delivery, placental abruption, acute lung edema, eclampsia, HELLP, or acute organ failure (41).

A high rate of FGR is particularly evident. The association of preeclampsia and placental insufficiency with consecutive fetal growth restriction has been reported previously. A common pathophysiological mechanism in remodeling of the spiral arteries during the development of the uteroplacental circulation is discussed (42). This theory describes changes before completion of placentation, mainly early-onset FGR occurs in patients with placental insufficiency, which is in agreement with our results, as most cases and especially the most severe cases of FGR were diagnosed before the 32nd gestational week. The cause of deficient remodeling is not known yet; however, oxidative stress, as well as immune, or endothelial factors are discussed, as natural killer cells seem to play an important role in the pathophysiology (43). Maternal cardiovascular pathologies might be a predisposition for that condition, as women with a history of cardiac diseases have a higher risk of developing placental insufficiency (44). Whether preeclampsia influences the cardiovascular profile of the fetus, in terms of embryonic imprinting, is currently unclear (45). According to the current literature, there is an association between the occurrence of preeclampsia or placental malperfusion and fetal congenital heart defects (46, 47). In our collective, four newborns were diagnosed with heart malformations of whom two required surgeries postnatally. The connection is certainly interesting and important, and the link between maternal and fetal hemodynamics is worth the effort to be investigated. However, it is not the focus of this work.

### Strengths and limitations

One of the main limitations of our study is its retrospective design, which inherently carries the risk of missing or incomplete data. Despite this, we demonstrated a significant association between NT-proBNP levels and both preeclampsia and preterm birth. However, we were unable to establish a cutoff value for NT-proBNP levels that could serve as a prognostic or predictive factor. Our study's findings must be interpreted in the context of notable limitations regarding missing data. Specifically, the TR Doppler signal, crucial for estimating PA pressure, was only measurable in 28% of our study population. This reflects daily clinical practice, where obtaining TR signals can be challenging, particularly in patients without overt cardiac pathology. Furthermore, while VCI was primarily used to assess RVP, we recognize the potential impact of uterine compression on VCI measurements in our pregnant study cohort. Additionally, the E/e' ratio data were notably limited, partly because tissue Doppler imaging has historically been employed selectively, primarily to resolve uncertainties regarding diastolic function or filling pressures in cases of doubt. Increased afterload, including hypertension, might yield underestimations of GLS. While direct blood pressure measurements were not recorded during echocardiographic evaluations, all participants were high-risk pregnant women under strict medical management to maintain normotension, which we assume was effective at the time of imaging. Our study employed RVOT VTI for stroke volume assessment, as per our institutional protocol, primarily to exclude pulmonary valve disease, acknowledging that this approach, though less common and potentially more error-prone than LVOT measurements, is supported by existing literature (48, 49). The LVOT VTI was measured selectively, based on specific clinical indications. This methodology reflects our institutional practice, but we recognize it as a limitation in terms of generalizability and comparability with more conventional approaches. In our study, the cardiac findings in pregnant women were benchmarked against reference values established for non-pregnant women. This approach, while useful for highlighting deviations from the norm, does not definitively distinguish between physiological adaptations to pregnancy and potential pathological changes. The lack of a control group comprising healthy pregnant women limits our ability to draw clear distinctions in this regard.

## Conclusion

Changes in cardiac function in the context of hypertensive pregnancy diseases can be observed concerning various echocardiographic parameters, including subclinical systolic dysfunction and elevated filling pressures. Additionally, a notable correlation exists between elevated NT-proBNP levels and reduced gestational age at delivery in women with preeclampsia. It is therefore advisable to perform early maternal echocardiography and measure proBNP when preeclampsia is diagnosed. This approach not only identifies early cardiac changes but also enables risk stratification based on proBNP levels, potentially enhancing prognostic accuracy for the timing of delivery and subsequent maternal and neonatal outcomes. While these insights are valuable for clinical application, currently there is no established cutoff for proBNP levels in pregnancy. Further research is essential to determine such a threshold to improve the predictive modeling of outcomes in preeclamptic pregnancies.

## Data Availability

The raw data supporting the conclusions of this article will be made available by the authors, without undue reservation.
