# Supplementary material for: Cardiac function in pregnant women with preeclampsia
Source: Front Cardiovasc Med. 2024 Dec 16;11:1415727. doi: 10.3389/fcvm.2024.1415727 (PMC11685740; doi:10.3389/fcvm.2024.1415727)
Supplement: Supplementary file 1 [file Table1.pdf]

Nr	**Indication for delivery**	**GA at birth**	**Birth weight (g)**	**APGAR**	**FGR**	**Survival time**	**Cause of neonatal death**
1	Severe preeclampsia, hypertensive crisis	23+5	490	7/9/9	Yes	3 days	Pulmonary hypertension, cardial decompensation
2	Severe preeclampsia, hypertensive crisis	23+6	370	6/8/9	Yes	4 days	Pulmonary hypertension, cardial decompensation
3	Severe preeclampsia, pathological fetal Doppler	26+4	573	8/9/9	Yes	30 days	NEC, sepsis
4	Maternal nephropathy, preeclampsia, pathological fetal Doppler	27+3	720	8/9/9	Yes	5 days	Persistent fetal circulation, pulmonary hypertension, sepsis
5	Severe preeclampsia	24+1	545	8/9/9	No	24 days	Cardial decompensation, sepsis, NEC and intestinal perforation

Supplementary Table 1 Cases of neonatal death in newborns delivered for maternal preeclampsia

GA: gestational age; FGR: fetal growth restriction;
